# Increased Oxidative Damage Associated with Unfavorable Cytogenetic Subgroups in Chronic Lymphocytic Leukemia

**DOI:** 10.1155/2014/686392

**Published:** 2014-06-26

**Authors:** Rosa Collado, David Ivars, Isabel Oliver, Carmen Tormos, Mercedes Egea, Amparo Miguel, Guillermo T. Sáez, Félix Carbonell

**Affiliations:** ^1^Service of Hematology, CDB-University General Hospital of Valencia, Avenida Tres Cruces 2, 46014 Valencia, Spain; ^2^Department of Medicine, Faculty of Medicine, University of Valencia, Avenida Blasco Ibáñez 13, 46010 Valencia, Spain; ^3^CIBERobn, Biomedical Network Research Centre in Physiopathology of Obesity and Nutrition, Choupana s/n, 15706 Santiago de Compostela, Spain; ^4^Department of Biochemistry and Molecular Biology, Faculty of Medicine, University of Valencia, Avenida Blasco Ibáñez 13, 46010 Valencia, Spain; ^5^Service of Clinical Analyses, CDB-University General Hospital of Valencia, Avenida Tres Cruces 2, 46014 Valencia, Spain

## Abstract

Oxidative stress contributes to genomic instability in chronic lymphocytic leukemia (CLL), but its relationship with the acquisition of specific chromosomal abnormalities is unknown. We recruited 55 untreated CLL patients and assessed 8-oxo-2′-deoxyguanosine (8-oxo-dG), glutathione, and malondialdehyde (MDA) levels, and we compared them among the cytogenetic subgroups established using fluorescence in situ hybridization (FISH). Significant increases in 8-oxo-dG and/or MDA were observed in patients with unfavorable cytogenetic aberrations (17p and 11q deletions) compared to the 13q deletion group. *TP53* deletion patients exhibited a diminished DNA repair efficiency. Finally, cases with normal FISH also showed enhanced 8-oxo-dG, which could result in adverse outcomes.

## 1. Introduction

Chronic lymphocytic leukemia (CLL) is the most common leukemia in Western countries and characterized by a variable clinical course, in which a portion of patients survive for long time periods without treatment, while others can experience early progression and subsequent death.

Based on fluorescence in situ hybridization (FISH) results, cytogenetic aberrations have been detected in 80% of patients with CLL. Patients with del(17p13) and del(11q22-23), which affect the* TP53* and* ATM* genes, respectively, have an unfavorable outcome followed by patients with trisomy 12. In contrast, patients with del(13q14), as the sole abnormality, exhibit prolonged survival times [1]. Recently, whole genome sequencing and improved culture techniques have provided additional information on novel recurrent alterations in CLL, such as mutations in* NOTCH1*,* SF3B1*,* MYD88*,* XPO1*, and* BIRC3 *genes,* IGH* translocations, and loss/gain of chromosome 8p/8q [2–4]. Interestingly, clonal evolution at the molecular level has been described in CLL. Tumor B-cells harbor different combinations of driver and passenger mutations that expand and evolve over time as response to changes in their local environment. This genetic diversity of cancer cells is essential for progression of the disease [5, 6].

On the other hand, accumulation of reactive oxygen species (ROS) results in a state of redox imbalance known as oxidative stress, which contributes to genomic instability. Previous analyses have demonstrated an impairment in the antioxidant defense system and an enhancement in the damaged DNA base 8-oxo-2′-deoxyguanosine (8-oxo-dG) in both the preleukemic state of monoclonal B-cell lymphocytosis and CLL [7, 8]. However, little is known regarding the relationship between the presence of oxidative damage and the acquisition of specific chromosomal aberrations. The aim of the present study was to assess different oxidative stress biomarkers in the context of the recurrent cytogenetic subgroups established using FISH in CLL.

## 2. Materials and Methods

### 2.1. Patients

The study included 55 untreated patients who were diagnosed with CLL according to IWCLL criteria [9]. This cohort was previously reported in Collado et al. [7] referring only to their oxidative stress status without relating to cytogenetic abnormalities pattern. All patients provided their written informed consent, and the study was performed according to the Helsinki declaration. The median age was 71 years (range: 47–92 years), and 89% of the subjects belonged to Binet stage A. The most important clinical and cytogenetic features of the studied patients are summarized in Table 1.

### 2.2. Blood and Urine Sampling

Heparinized whole blood was diluted with RPMI 1640 medium (Gibco BRL), and mononuclear cells were isolated by Lymphoprep (Nycomed) centrifugation. Cells were stored at −80°C until analysis. Twenty-four-hour urine samples were collected and homogenized. Then, urine samples were frozen at −20°C until use.

### 2.3. DNA Isolation and Enzymatic Digestion

Cellular DNA was isolated following the method in which chloroform isoamyl alcohol (24 : 1) was used instead of phenol to remove proteins. Isolated DNA was washed twice with 70% ethanol, dried, and dissolved in 200 *μ*L of 10 mM Tris-HCl, 0.1 mM EDTA, and 100 mM NaCl (pH 7.0) for enzymatic digestion. To this end 5 *μ*g of DNA/*μ*L (total DNA content 200 *μ*g) was incubated with 100 units of DNase I in 40 *μ*L of Tris/HCl (10 mM) and 10 *μ*L of 0.5 M MgCl_2_ (final concentration: 20 mM) at 37°C for 1 h. The pH of the reaction mixture was then lowered with 15 *μ*L of 0.5 M sodium acetate (pH 5.1); 10 *μ*L of nuclease P1 (5 units) and 30 *μ*L of 10 mM ZnSO_4_ were added to give a final concentration of 1 mM, and the mixture was incubated for 1 h. After adjusting the pH with 100 *μ*L of 0.4 M Tris/HCl (pH 7.8), followed by addition of 20 *μ*L of alkaline phosphate (3 units), the samples were incubated for 30 min. Enzymes were precipitated with acetone (5 volumes) and removed by centrifugation, and the supernatant was evaporated to dryness.

### 2.4. Lymphocyte and Urinary 8-oxo-dG Assay

The DNA hydrolysates were dissolved in HPLC grade water and filtered through a 0.2 mm syringe filter before the samples were applied onto a Waters ODS HPLC column (2.5 × 0.46 i.d.; 5 *μ*m particle size). The amounts of 8-oxo-dG and dG in the DNA digest were measured by electrochemical and UV absorbance detection, respectively, under the appropriated elution conditions. Standard samples of dG and 8-oxo-dG were analyzed to assure their good separation and to allow the identification of those moieties derived from cell DNA. To carry out the detection of urinary 8-oxo-dG, we followed the method described by Collado et al. [7]. The 8-oxo-dG values were expressed as ratios to urine creatinine concentrations given in mmol/mL.

### 2.5. Glutathione and Lipid Peroxidation Assay

Glutathione (GSH) content of cells was measured according to the method of Brigelius et al. [10]. Malondialdehyde (MDA) levels were analyzed by HPLC. Briefly, the technique employs the hydrolysis of plasma lipoperoxides by boiling in dilute phosphoric acid. MDA is one of the hydrolysis products and reacts with thiobarbituric acid (TBA) to form MDA(TBA)2 adduct, and plasma proteins are precipitated with methanol and removed from the reaction mixture by centrifugation. The resulting extract is fractionated by HPLC on a column of silica gel, to separate the MDA-TBA adduct, which finally is eluted with methanol/phosphate buffer and quantified spectrophotometrically at 532 nm.

### 2.6. Fluorescence In Situ Hybridization

Interphase FISH analyses were performed on fixed cells obtained from 72 h peripheral blood cultures stimulated with phorbol 12-myristate 13-acetate (TPA). The following probes were used: LSI* ATM*/CEP11, CEP12, LSI* D13S319*/LSI13q34, and LSI* TP53*/CEP17 (Abbot Co., Des Plaines, IL). Hybridization signals were evaluated under a fluorescence microscope (Eclipse E800, Nikon) equipped with a charge-coupled device (CCD) camera run by Cytovision software (Applied Imaging, Newcastle upon Tyne, UK). The number of interphase nuclei analyzed was 200 per sample. The cut-off values were 5% for CEP12 probe and 10% for LSI* ATM*/CEP11,* D13S319*/13q34, and* TP53*/CEP17 probes.

### 2.7. Statistical Analysis

The normal distribution was tested using the Shapiro-Wilk test. One-way analysis of variance (ANOVA) and Student's *t*-test were used to analyze differences among groups. Results are expressed as mean ± standard deviation (SD). The statistical package SPSS, version 15.0 (SPSS Inc., Chicago, IL, USA), was used for all analyses. A two-sided *P* value ≤0.05 was considered significant.

## 3. Results and Discussion

CLL demonstrates a highly variable disease course, which is partly explained by the process of clonal evolution. Moreover, antioxidant and oxidative stress variations greatly influence genetic and epigenetic cascades underlying altered gene expression in human carcinogenesis [11]. However, the effect of oxidative stress levels on cytogenetic heterogeneity in CLL is unknown. This study included a cohort of 55 untreated patients. Interphase FISH analysis revealed eighteen patients with 13q14 deletion as the sole abnormality, eight patients with trisomy 12, six patients who presented deletion of* ATM* gene at 11q22-23, and six patients who harbored 17p13 deletion affecting the* TP53* gene. In seventeen cases, no abnormality was detected using the 4-probe panel. We measured the most representative oxidative stress parameters, such as 8-oxo-dG, and glutathione and malondialdehyde (MDA) levels in B-cells and urine from CLL patients and compared these values among the cytogenetic subgroups (Table 2).

**Figure 1:**
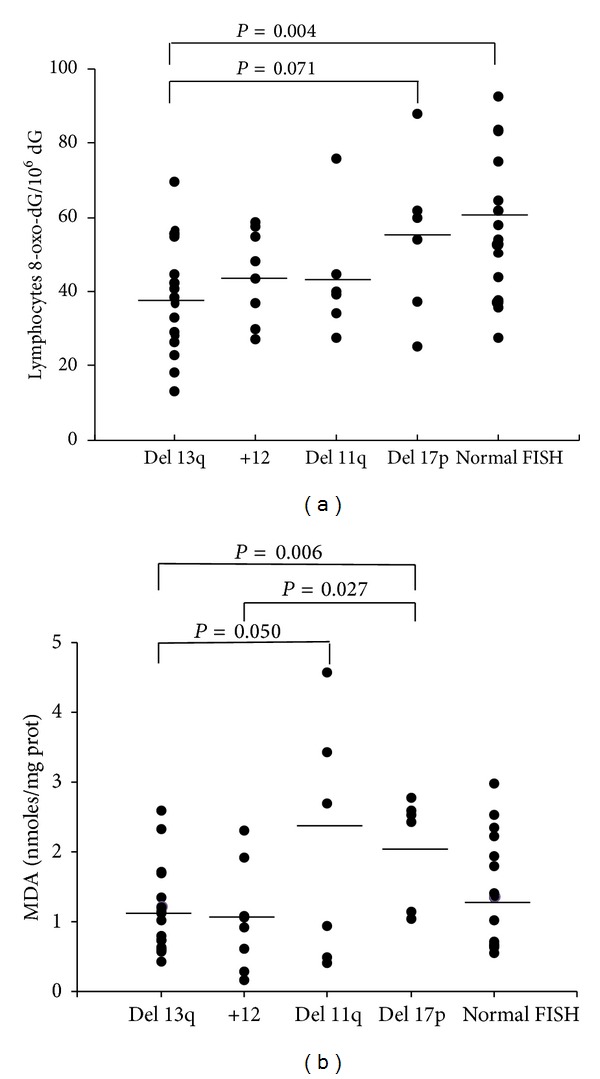
Analysis between cytogenetic subgroups with respect to (a) lymphocytes 8-oxo-dG/10^6^ dG levels and (b) malondialdehyde-MDA levels. The mean value is shown for each group (*bars*). *P* values were determined on the basis of the Student's *t*-test.

Increasing knowledge regarding the use of new biomarkers in the early detection and followup of cancer has attracted attention within the last years. Repair of modified guanine prior to DNA replication is an essential feature for the maintenance of cell homeostasis because 8-oxo-dG is a mutagenic base that can result in GC→TA transversions. The frequency of mutations generated by 8-oxo-dG presence in mammalian cell DNA is 2.5–4.8%; therefore, increased levels of 8-oxo-dG may contribute to gene instability, affecting the normal function of oncogenes and tumor suppressor genes [12]. In lymphocytes from CLL patients, we detected global significant differences (*P* = 0.034) compared to the 8-oxo-dG levels among the five cytogenetic groups assessed. Interestingly, patients with a deletion in* TP53* and the normal FISH group showed higher concentrations of the damaged base (54.46 ± 21.66 and 57.17 ± 18.45 8-oxo-dG/10^6^ dG, resp.). As shown in Figure 1(a), significant increases in 8-oxo-dG level were observed in the normal FISH group compared with the favorable prognostic 13q deletion group (57.17 ± 18.45 versus 39.40 ± 15.69 8-oxo-dG/10^6^ dG, *P* = 0.004). Similarly, we detected a trend towards significance (*P* = 0.071) when comparing patients with* TP53* and 13q deletions, which might be due to the small number of cases in the* TP53* deletion group. It has been widely established that activated p53 is involved in growth arrest, DNA repair, apoptosis, and senescence pathways. However, Sablina and colleagues [13] have recently postulated a new role for p53, suggesting that p53 may extend its protective function by participating in antioxidant defense. Genes such as* SOD2*,* GPX1*,* ALDH4A*,* HI95*, and* PA26* encode products that act as antioxidants and are modulated by p53. Moreover, downregulation of p53 elevates intracellular ROS, thereby increasing the mutation rate and karyotype instability. In these sense, Macedo et al. [14] also described increased oxidative damage in carriers of the germline* TP53* p.R337H mutation in families with Li-Fraumeni syndromes. Our data were consistent with these findings, where defective p53 derived from* TP53* deletion in CLL cells resulted in an increased level of 8-oxo-dG. The occurrence of important oxidized DNA base concentrations among patients with deletions and/or mutations of* TP53 *in CLL may explain the poor prognosis observed in these cases, in addition to the involvement of* TP53* alterations in complex karyotypes [15, 16]. Furthermore, the observed DNA damage in the normal FISH group could contribute to the emergence of karyotypic aberrations with adverse prognostic features that have been previously described by Rigolin et al. [17] in one-third of CLL patients with normal FISH. In fact, two of our cases carried karyotypic abnormalities in regions not covered by the 4-probe FISH panel used. One case harbored a deletion del(12)(p12), and the other one showed a translocation t(2;7)(p21;q32).

On the other hand, 8-oxo-dG is present in the urine as a consequence of the base/nucleotide excision repair pathways [12]. Although no significant differences were found among urinary 8-oxo-dG levels in the present study, it is noteworthy that patients with* TP53* deletion exhibited lower concentrations (12.22 ± 4.50 nmol/mmol creatinine), suggesting a diminished DNA repair efficiency in this group. The mechanism underlying this decrease is still not known but is most likely due to an alteration in* OGG1 *gene expression [12]. An additional important element in the antioxidant defense is ROS detoxification via the activation of glutathione peroxidase and elevation of thiols. Glutathione is an antioxidant intracellular thiol that is increased in circulating blood cells of CLL patients [7, 8]. However, we have not observed any differences among the analyzed cytogenetic subgroups. Likely, increases in the GSH levels were not sufficient to prevent oxidative stress in CLL cells.

Finally, lipid peroxidation may cause significant changes in the functional properties of biomembranes, resulting in altered cell signaling and in the release of toxic cellular products. In this study, we used MDA as a marker of lipid peroxidation. Differences in MDA levels among cytogenetic groups reached global statistical significance (*P* = 0.05). Thus, we observed an association between plasma MDA content and poor prognosis genetic subgroups. CLL patients with* TP53* and* ATM* deletions demonstrated significantly higher MDA levels than patients with 13q deletions and trisomy 12 (Figure 1(b)). These results confirmed the findings observed by Macedo et al. [14] in* TP53* p.R337H carriers. In addition, Shiloh and Ziv [18] have recently proposed a role of* ATM* in regulating the cellular redox balance, indicating its effects on mitochondrial physiology.* ATM*-deficient cells have been associated with mitochondrial dysfunction. Consistent with this finding, lipid peroxidation observed in CLL patients with* ATM* deletions may disturb the mitochondrial membrane potential, thereby affecting its normal activity.

## 4. Conclusions

Taken together, our results showed that B-cells from CLL patients with unfavorable cytogenetic aberrations were susceptible to injury caused by ROS, resulting in increased levels of oxidized DNA bases as 8-oxo-dG and/or lipid peroxidation. Because a portion of patients with CLL acquire new aberrations during the course of the disease, we presumed that a deficiency in the* TP53* tumor suppressor gene could promote oxidation-mediated mutagenesis, thereby contributing to accelerated malignant progression. Moreover, attention must be focused on mutations resulting in altered activity of DNA repair mechanisms as well as polymorphisms of DNA repair genes that contribute to resistance to treatment with alkylating agents, such as chlorambucil, or with the purine analog fludarabine. Moreover, further studies are required to elucidate the genetic content of CLL patients with normal FISH. The occurrence of the new unfavorable* NOTCH1, SF3B1, and BIRC3* mutations in this patient group is an important issue that we have to analyze in the future.

Lastly, the present study supports the possibility of using antioxidants in combination with existing therapeutic strategies to improve survival in CLL patients. In this line, Jitschin et al. [19] have recently treated CLL cells with the antioxidant N-acetyl-cysteine (NAC). Oxidative stress attenuates immune responses by leading to dysfunctions and even apoptosis of NK- and T-cells, suggesting a role in tumor immune escape. The addition of NAC prevented CLL cell-mediated T-cell dysfunction. The authors concluded that treatment of CLL cells using antioxidants could neutralize endogenous ROS and protect the immune system. Such strategies have been successfully implemented in other hematological entities such as acute myeloid leukemia [20].

## Figures and Tables

**Table 1:** Summary of clinical and cytogenetic characteristics of the CLL cases included in the present study.

Patient characteristics (*n* = 55)	
Age (years)	71 (47–92)
Male	30 (54.5%)
Binet stage	
A	49 (89.1%)
B	5 (9.1%)
C	1 (1.8%)
White blood cell count (×10^9^/L)	28.6 (7.30–240.0)
Lymphocyte count (×10^9^/L)	23.2 (5.6–233)
Hemoglobin (g/L)	13.8 (9.1–16.8)
Platelet count (9109/L)	189.0 (20.0–373.0)
Lactate dehydrogenase (U/L)	358.0 (115–1086.0)
*β*2-Microglobulin (mg/L)	2.3 (1.0–6.54)
ZAP70 positive∗ (*n* = 37)	22 (59.4%)
CD38 positive∗ (*n* = 45)	12 (26.6%)
FISH abnormalities	
del(13q14)	18 (32.7%)
Trisomy 12	8 (14.5%)
del(11q22)	6 (11.0%)
del(17p13)	6 (11.0%)
Normal FISH	17 (31.0%)

Values are given as median (range) or number (%).

*Positivity was considered when ZAP70 > 20% and CD38 > 30%.

**Table 2:** Oxidative stress parameters and cytogenetic subgroups in CLL patients.

	del(13q14) *n* = 18 Mean ± SD	Trisomy 12 *n* = 8 Mean ± SD	del(11q22) *n* = 6 Mean ± SD	del(17p13) *n* = 6 Mean ± SD	Normal FISH *n* = 17 Mean ± SD	*P* value^a^
Lymphocytes 8-oxo-dG/10^6^ dG	39.40 ± 15.69	44.65 ± 12.26	43.58 ± 16.82	54.46 ± 21.66	57.17 ± 18.45	**0.034**
Urinary 8-oxo-dG (nmol/mmol creatinine)	19.70 ± 15.32	22.87 ± 15.13	27.31 ± 15.03	12.22 ± 4.50	18.76 ± 11.60	0.676
Glutathione (nmol/mg prot.)	28.28 ± 5.08	26.15 ± 5.75	35.48 ± 9.00	26.72 ± 6.35	28.30 ± 8.09	0.118
MDA (nmol/mg prot.)	1.14 ± 0.61	1.04 ± 0.74	2.09 ± 1.73	2.08 ± 0.78	1.48 ± 0.78	**0.050**

8-oxo-dG: 8-oxo-2-deoxyguanosine; MDA: malondialdehyde; SD: standard deviation.

^a^To determine whether there were any global significant differences between the means of cytogenetic subgroups, we performed the one-way analysis of variance (ANOVA) test.
